# The possible effect of forceful eye closure and eye opening on nasal valve cross-sectional area during endoscopy

**DOI:** 10.1007/s00405-026-10103-4

**Published:** 2026-03-15

**Authors:** Alisa Luria, Keren Oren, Yehuda Schwarz, Oded Cohen, Ofer Gluck

**Affiliations:** Department of Otolaryngology-Head and Neck Surgery, Faculty of Health Sciences, Samson Assuta Ashdod University Hospital, Ben Gurion University, 7, Ha’Refua Street, 7747629 Ashdod, Israel

**Keywords:** Nasal endoscopy, Nasal valve, Patient comfort, Eye closure, Endoscopic technique

## Abstract

**Background:**

Nasal endoscopy is a routine procedure in the otolaryngology clinic, yet intra-procedural discomfort is common and may deter future examinations. Preliminary observations suggest that eye closure may affect the nasal valve cross-sectional area, which, in turn, has the potential to impact discomfort during endoscopy, as it is the narrowest part of the nasal passageway.

**Objective:**

To evaluate the effect of forceful eye opening and closure on the nasal valve’s cross-sectional area using rigid video-endoscopy and digital image analysis.

**Methods:**

A prospective pilot case series study. Nasal video endoscopy of the right nasal vestibule was performed by a rigid endoscope in 3 facial positions: relaxed, eyes tightly closed, and eyes wide open. Nasal valve images were assessed by two independent, blinded examiners, who quantified the nasal valve cross-sectional area. The area size in the “relaxed” position was used as a reference against which the area sizes in other facial positions were normalized.

**Results:**

The study included 36 volunteers, of which 27 were females (75%). The mean age was 40.7 ± 12.7 years. The nasal valve cross-sectional area was significantly smaller at the “eyes tightly closed” position, compared with the “relaxed” position (Z = -5.232, p-value < 0.001) and with the “eyes wide open” position (Z = -5.169, p-value < 0.001). No significant difference was found between the “relaxed” and “eyes wide open” positions.

**Conclusion:**

This hypothesis-generating pilot study demonstrated that tight eye closure is associated with a significant decrease in the cross-sectional area of the nasal valve. Considering the possible relation between a narrow passage and discomfort, further studies investigating the association between eye closure and patients’ discomfort during endoscopy are encouraged.

**Supplementary Information:**

The online version contains supplementary material available at 10.1007/s00405-026-10103-4.

## Introduction

Passage of the endoscope in the nasal cavity is an essential part of the otolaryngological physical examination, as well as other procedures such as transnasal bronchoscopy and transnasal esophagogastroduodenoscopy [1–5]. Despite its routine use in various fields, many patients experience discomfort and even pain during the procedure [6–10]. While many patients consider the discomfort to be minor, others may experience more significant pain, which could lead to avoidance of future examinations.

Nasal breathing has been shown to alleviate pain during transnasal endoscopy [11], suggesting that certain behavioral adjustments during the examination may provide additional means to decrease discomfort. It is not uncommon for patients to close their eyes during a nasal endoscopy as a reaction to pain or under the assumption that looking at the procedure may induce or aggravate anxiety. Our clinical observation has been that patients who closed their eyes during examination had a narrower nasal passage and experienced increased discomfort. Following that, the authors were driven to explore the impact of facial expression on the cross-sectional area of the nasal valve, which is the narrowest point in the nasal passageway.

The authors hypothesized that forceful eye closure or opening would affect the cross-sectional area of the nasal valve. To the best of our knowledge, the current study is the first to investigate this association. While this is a hypothesis-generating pilot study, such an association may encourage future studies focusing on behavioral measures to reduce endoscopy-induced discomfort.

## Materials and methods

### Study design and participant selection

This is a prospective pilot case series study. The study was approved by the institutional review board. Participants included in the study were adult patients visiting the otolaryngology clinic between March and June 2024 for non-rhinologic complaints who consented to participate. Exclusion criteria included: pediatric patients (< 18 years), pregnancy, prior facial or nasal surgery or treatment including facial injections (Botox, collagen, etc.), history of nasal trauma, severe nose deformation, nasal tumors or facial anomalies, caudal deviation of the septum, neuromuscular or connective tissue disorders, known inflammatory nasal conditions including chronic usage of local or systemic steroids (> 3 months), chronic nasal decongestant use, and nasal complaints (i.e. nasal obstruction, rhinorrhea, hyposmia). All patients who consented to participate were interviewed by the authors to exclude these criteria.

### Endoscopic nasal valve measurement

Based on methods employed in previous studies [12–14], video-endoscopy of the anterior nasal airway and digital image analysis were used to assess the nasal valve cross-sectional area. Each participant’s nasal valve endoscopic images were recorded in three positions: (a) a relaxed face, (b) eyes tightly closed, and (c) eyes wide open. Only the right nasal valve was assessed in each participant in order to standardize the examination and reduce variation. The primary outcome of the study was the nasal valve cross-sectional area, calculated as detailed below.

**Fig. 1:**
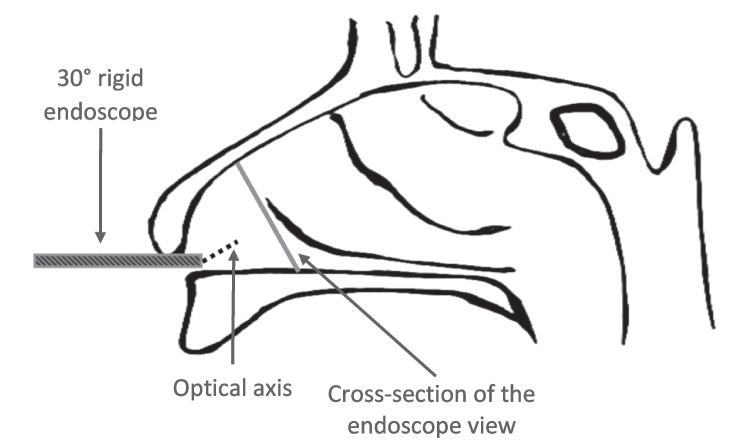
An illustration demonstrating the study’s set-up of the nasal endoscopy using a 30° rigid endoscope. The optical axis and the cross-section of the endoscope view are marked. [13] Adapted from Keck et al.

All endoscopic recordings were performed by the first author (A.L) in the same examination room. Participants were relaxed, refrained from any exercise for at least 90 min before testing, and were acclimatized to a 22 °C air-conditioned room. All measurements were conducted without the influence of nasal decongestants or anesthetics. The endoscopic recording and image analysis procedures were adapted from Keck et al. [13]: a rigid endoscope (7230 BA, HOPKINS® Forward-Oblique Telescope 30°, 4 mm diameter, Karl Storz) equipped with a video recorder was used to capture and record videos for all endoscopy exams. A video sequence was recorded with the endoscope positioned on the floor of the right nasal vestibule 5 mm beyond the nostril entrance. The set-up of the nasal endoscopy is presented in Fig. 1. During the recording, the participants were sequentially instructed to (a) remain with a relaxed facial expression, (b) close their eyes tightly, and (c) open their eyes wide, remaining in each position for two seconds. The three facial positions were recorded in one sequence without a rest period between them. The participants were instructed to avoid inhaling or exhaling during the recording to eliminate the possible effect of nasal breathing on the nasal valve patency and reduce movements during video-recording. In addition to the collection of the endoscopy recordings, each participant’s sex and age were documented.

**Fig. 2:**
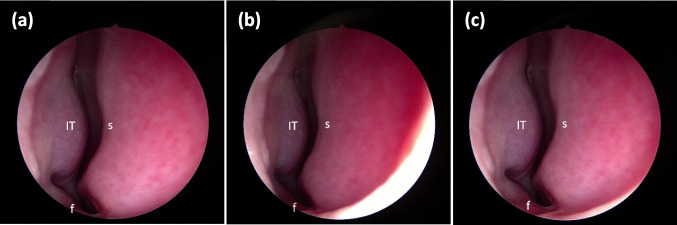
Examples of images of a patient’s nasal valve with (**a**) a relaxed face, (**b**) eyes tightly closed and (**c**) eyes wide open. The anatomic landmarks are marked in each image: *f* – nasal floor, *s* – septum, and *IT* – head of inferior turbinate

**Fig. 3:**
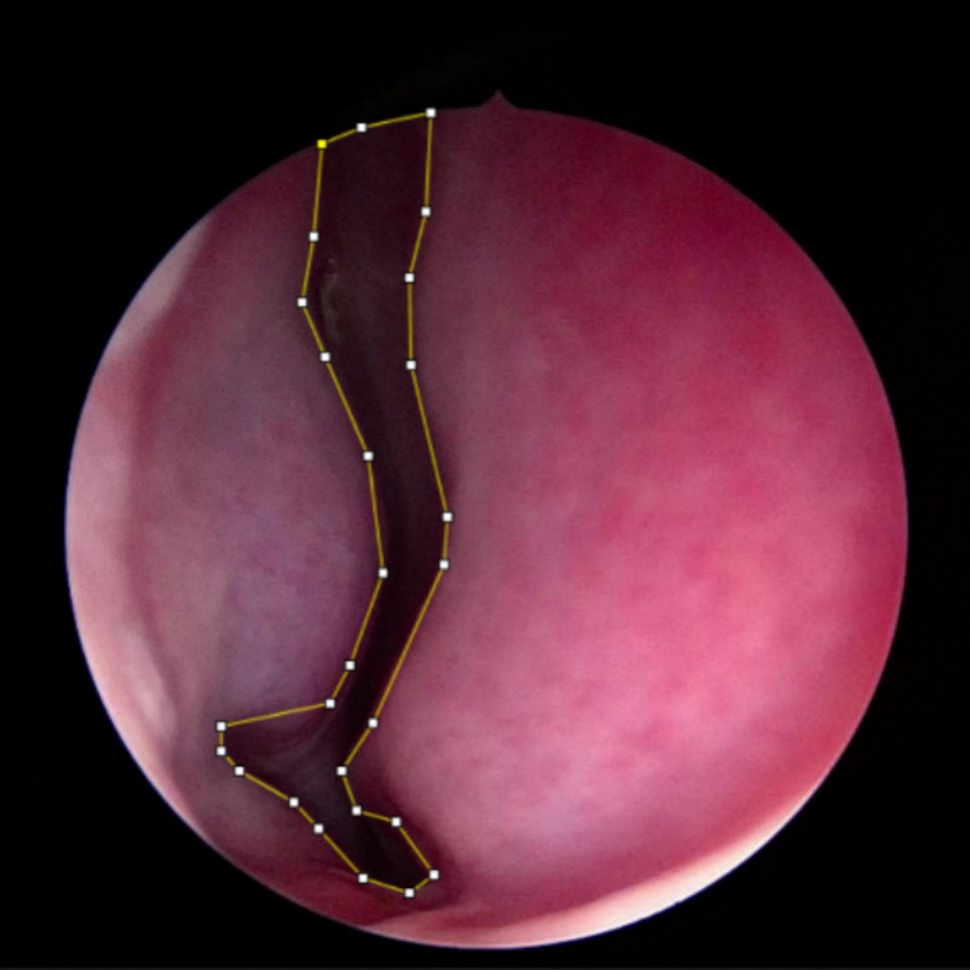
An example of a nasal valve image with the outline of the nasal valve cross-sectional area marked for automatic area calculation in ImageJ software

All recordings were transferred to a laptop and digitally analyzed. Three images from the recorded video sequence, one from each position (a)-(c), were captured. The images were selected from the time period after the corresponding instruction and when the following landmarks were visible without obstruction: nasal floor, septum, and head of inferior turbinate. Examples of images captured in each position with the anatomical landmarks marked are presented in Fig. 2. Digital image analysis was performed by two independent examiners (K.O. and O.G.), blinded to each other and to the test condition of each image. The examiners used ImageJ open‐source image processing and analysis software [15] to measure the cross-sectional area of the nasal valve in each image by marking its outline on the image. An example of a captured nasal valve image with the outline of the nasal valve cross-sectional area is presented in Fig. 3.

### Data storage and safety

All experimental procedures were explained in full detail to the study participants, who provided written informed consent. Each participant was given a serial number, and the endoscopy recording was saved without any identifying details using that number. A password-protected file was created, available only to the examining physician, containing each participant's ID number, serial number given, sex and age.

### Statistical analysis

Data were collected into a spreadsheet, and statistical analysis was performed with IBM SPSS Statistics (Version 23, 2015, Armonk, NY).

Intraclass Correlation Coefficient (ICC) was calculated between the two raters for each condition. ICC estimates and their 95% confident intervals were calculated based on a mean-rating (k = 2), absolute-agreement, 2-way random-effects model. All three conditions demonstrated good inter-rater agreement (ICC ≥ 0.6), with the “relaxed” showing excellent agreement (ICC ≥ 0.75; Table 1). As the ICC was sufficiently high for all conditions, average values across raters were used for all analyses.

**Table 1:** Inter-rater ICC estimates and their 95% confident intervals

Condition	Average Measures	95% Confidence Interval	F statistic	*p*-value
Eyes Relaxed	0.753	(0.304,0.786)	F_35,35_ = 4.773	< 0.001
Wide Open	0.689	(0.342,0.848)	F_35,35_ = 3.811	< 0.001
Tightly Closed	0.738	(0.487,0.867)	F_35,35_ = 4.105	< 0.001

Relaxed facial position was defined as the reference point for both eyes wide open and eyes tightly closed positions, which allowed normalization for each subject, better representing the relations between the different measurements (as compared to absolute measurements of the nasal valve regardless of a reference point). For this purpose the authors defined these three conditions: (1) normalized relaxed (defined as $100\times \frac{{Relaxed}_{i}}{{Relaxed}_{i}},$); (2) normalized open (defined as $100\times \frac{{Open}_{i}}{{Relaxed}_{i}},$) and (3) normalized closed (defined as $100\times \frac{{Closed}_{i}}{{Relaxed}_{i}},$). The normalized data were used for all following analyses.

Data were assessed for normality per paired differences using the Shapiro–Wilk test (Table S1). Only the “relaxed” vs “eyes tightly closed” difference passed the normality test. However, the corresponding histogram (Figure S1) indicated deviation from normality. The other paired differences did not pass the normality test. For consistency’s sake, following analyses were conducted via non-parametric tests.

Following a statistically significant Friedman test (F_2_ = 35.389, *p*-value < 0.001), pairwise Wilcoxon tests with adjustments for multiple comparisons using Bonferroni’s correction were used to test whether a difference in nasal valve cross-sectional area exists between the different conditions. Effect size was measured using Hodges-Lehmann median differences test. Difference between males and females was assessed using the Mann–Whitney U Test.

## Results

**Fig. 4:**
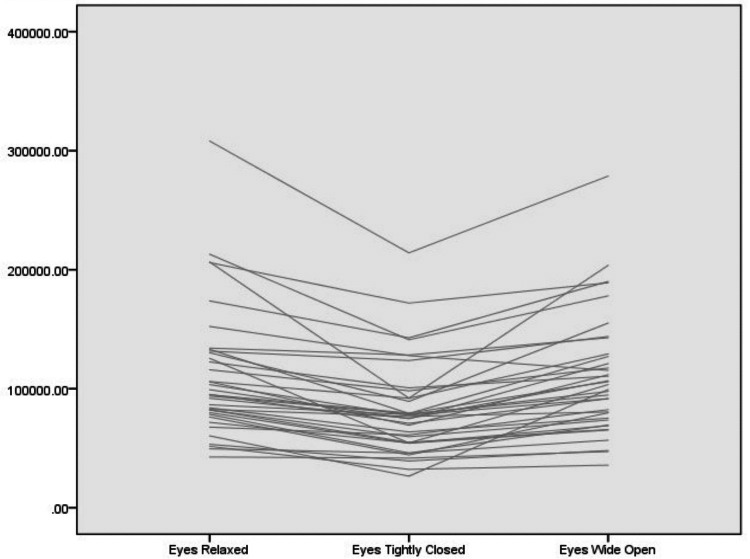
In-subject spaghetti plot of the nasal valve cross-sectional area in different positions

The study included 36 participants, of which 27 (75%) were female. Mean age was 40.7 ± 12.7 years. Participant demographics and measurements of nasal valve cross-sectional areas by both examiners are presented in Table 2, and an individual-subject spaghetti plot is presented in Fig. 4. Descriptive statistics of the normalized data are provided in Table S2.

**Table 2:** Participants’ demographic details and measurements of nasal valve cross-sectional area in three positions: a relaxed face, eyes tightly closed, and eyes wide open

Participant number	Age	Sex	Measured nasal valve cross-sectional area (px)					
			Examiner 1			Examiner 2		
			Relaxed face	Eyes tightly closed	Eyes wide open	Relaxed face	Eyes tightly closed	Eyes wide open
1	32	F	89944	60365	77743	77857	66923	72698
2	30	F	98839	74921	99979	88805	82915	89323
3	34	F	153469	145177	160059	114744	111336	125685
4	53	F	147443	122444	139418	84331	73968	95358
5	33	M	191863	167148	215246	155673	118271	164879
6	35	F	99417	71538	102131	84491	78507	111174
7	28	F	81099	60620	67367	78663	47850	63201
8	40	M	61607	59516	94660	73783	62774	70184
9	33	F	125433	79379	107922	64972	62446	75320
10	26	F	108003	96668	129910	104155	87254	128482
11	51	M	84117	57174	77588	80168	62082	69732
12	53	F	52880	42987	47838	53671	35608	48173
13	41	F	127188	94150	130081	177481	161411	100638
14	22	F	211803	101344	276050	201153	82637	131371
15	31	F	140919	86548	123171	70569	52172	100279
16	29	M	114761	75888	103562	92183	79266	79766
17	30	F	51244	50057	53988	47686	41823	59393
18	27	F	406252	277606	383442	209948	150682	173957
19	62	M	223852	202206	202432	39320	45037	85318
20	53	F	156312	63352	110468	94662	45436	96329
21	50	M	191131	107957	186827	74923	50473	67264
22	60	F	46949	33640	39363	56097	30757	32072
23	54	F	86441	77565	97627	86634	78772	97908
24	48	F	88119	51013	75627	68629	40798	63297
25	33	M	85705	56797	65924	78573	52879	65682
26	41	F	80948	48649	81389	71382	39951	78142
27	27	F	78754	50585	73578	64628	58265	64148
28	59	F	81723	81048	90067	83510	72311	70905
29	53	F	60185	40028	133055	60481	12946	65583
30	68	M	44463	36316	55606	40760	46997	38686
31	53	F	223496	194039	219475	188446	149781	158470
32	20	F	95225	86398	112505	94512	72532	99393
33	34	F	125733	84519	165415	134679	94187	144950
34	36	F	108987	74369	130093	89420	75192	112108
35	51	M	113297	80510	99345	131599	120812	121686
36	35	F	219453	125373	130500	206577	156877	225610

The cross-sectional area of the nasal valve was found to be 24.381 normalized units smaller (Z = −5.232*, p*-value < 0.001) in the “eyes tightly closed” position compared to the “relaxed” position, and 25.682 normalized units smaller (Z = −5.169*, p*-value < 0.001) in the “eyes tightly closed” position compared to the “eyes wide open” position. No statistically significant difference was found in the nasal valve cross-sectional area between the “relaxed” and “eyes wide open” positions (Z = −0.063,* p*-value = *0.950)*. The Hodges-Lehmann estimator of the median of differences is 24.21 (95% CI = 19.41 to 29.24, *p*-value < 0.001) for “eyes tightly closed” position compared to the “relaxed” position, and 22.83 (95% CI = 17.39 to 28.84* p*-value < 0.001) for the “eyes tightly closed” position compared to the “eyes wide open” position (Table 3).

**Table 3:** Results of pairwise Wilcoxon tests and Hodges-Lehmann median differences (normalized units)

Condition	Wilcoxon test				Hodges-Lehmann Median Differences			
	Median Difference	Statistic	*p*-value	Bonferroni corrected *p*-value	Hodges-Lehmann estimate	95% CI	*p*-value	Bonferroni corrected *p*-value
Relaxed vs Closed	24.381	Z = −5.232	< 0.001	< 0.001	24.21	(19.41, 29.24)	< 0.001	< 0.001
Open vs Closed	25.682	Z = −5.169	< 0.001	< 0.001	22.83	(17.39, 28.84)	< 0.001	< 0.001
Open vs Relaxed	1.956	Z = −0.063	0.95	1	−2.36	(−8.25, 5.65)	0.6691	1

No statistically significant difference was detected between males and females with respect to the normalized nasal valve cross-sectional area in either “eyes tightly closed” (Z = 0.932, *p*-value = 0.352) or “eyes wide open” positions (Z = 0.311, *p*-value = 0.756) (Table S3).

## Discussion

The aim of the current study was to evaluate the effect of forceful eye opening and tight eye closure on the nasal valve cross-sectional area. Our results show that tight eye closure causes a significant reduction in the nasal valve cross-sectional area.

Several mechanisms may explain the observed association between eye closure and reduced nasal valve cross-sectional area. One possible explanation is the associated innervation of the Orbicularis Oculi muscle and the nasal valve musculature. Anatomically, nasal valve musculature is largely innervated by the buccal branch of the facial nerve. Guyuron described the Depressor Alae, Levator Labi Superioris, and Pars Alaris (Nasalis) muscles as related to the external nasal valve [16], while Bruintjes et al. identified the role of the Dilator Naris, Alar Nasalis, and Transverse Nasalis muscles in stabilizing and opening the external valve [17]. Meanwhile, the Orbicularis Oculi muscle is innervated by the zygomatic and temporal branches of the facial nerve. Notably, communications between the zygomatic and buccal branches occur in approximately two-thirds of individuals [18, 19]. Another potential explanation is that the contraction of the Orbicularis Oculi mechanically influences the nasal valve muscles. Strong eyelid closure activates the outer (orbital) part of the Orbicularis Oculi muscle, and fibers in this peripheral part overlap with fibers of other muscles, including the nasal muscles [20, 21].

While this pilot study focuses on the effect of eye closure and opening on nasal anatomy, it may serve as a starting point for future studies exploring whether the observed change in nasal valve cross-sectional area has a clinical implication on patient discomfort during nasal endoscopy. This hypothesis is based on a study by Takahashi et al. [11] which laid the ground associating between resistance to endoscope passage and discomfort. They demonstrated that nasal breathing during transnasal endoscopy resulted in reduced patients’ discomfort and pain compared to oral breathing. The suggested mechanism behind this phenomenon was that during nasal breathing, the soft palate moves downward to the root of the tongue, resulting in a patent upper-middle pharynx and lesser resistance to endoscope passage. It is yet to be assessed where exactly contact and pain occur during nasal endoscopy. For example, it may result from the endoscope touching the septum or creating friction and traction on the soft triangle of the nose. Therefore, we suggest that future research should investigate whether instructing patients to avoid forceful eye closure can reduce endoscope contact with the nasal mucosa and decrease patient discomfort during nasal endoscopy.

The effect of other factors on the nasal valve cross-sectional area should be considered as well and explored in future studies. Firstly, the nasal valve cross-sectional area may be affected by the patient’s use of other facial muscles during nasal endoscopy. Moreover, nasal valve collapse could result from pathologies and post-surgical conditions which were excluded from this study, such as weakened or lacking cartilage from rhinoplasty, caudal septal dislocation, trauma, lax connective tissue due to the aging process [22], or facial paralysis [23, 24].

Our research is a pilot study with a limited sample size. Due to time constraints and participants’ comfort considerations, the three facial positions were tested sequentially in a fixed order (relaxed, eyes tightly closed, eyes wide open) without a rest period in between, and only the right nasal valve was assessed in all participants. The lack of order randomization and single-nostril testing invites order effects and reduces generalizability. In this study, the cross-sectional nasal valve area was calculated using a two-dimensional image, which is an imperfect estimate of the minimal cross-sectional area. An additional limitation is the possibility of a human error in measuring the cross-sectional nasal valve area, even by a blinded examiner. Future studies may benefit from using artificial intelligence tools to analyze images in order to eliminate observer bias. Finally, the study conditions differed from common clinical endoscopy settings in that the participants were non-rhinologic patients tested without decongestant or anesthetic. Although the listed limitations constrain generalizability, the association between eye closure and nasal surface area found in the study supports further research.

## Conclusion

In this pilot study, forceful eye closure was associated with a within-subject reduction in endoscopic estimates of nasal valve cross-sectional area. While hypothetical, it may support future trials that would test whether instructing patients to keep their eyes open measurably reduces endoscope-mucosa contact and patient-reported discomfort during nasal endoscopy.

## Supplementary Information

Below is the link to the electronic supplementary material.

Supplementary file1 (PDF 5376 KB)
