# Supplementary material for: The possible effect of forceful eye closure and eye opening on nasal valve cross-sectional area during endoscopy
Source: Eur Arch Otorhinolaryngol. 2026 Mar 15;283(5):3121–7. doi: 10.1007/s00405-026-10103-4 (PMC13152924; doi:10.1007/s00405-026-10103-4)
Supplement: Supplementary file 1 — Supplementary file1 (PDF 5376 KB) [file 405_2026_10103_MOESM1_ESM.pdf]

The Possible Effect of Forceful Eye Closure and Eye Opening on Nasal Valve Cross-sectional Area During Endoscopy

Supplementary Information

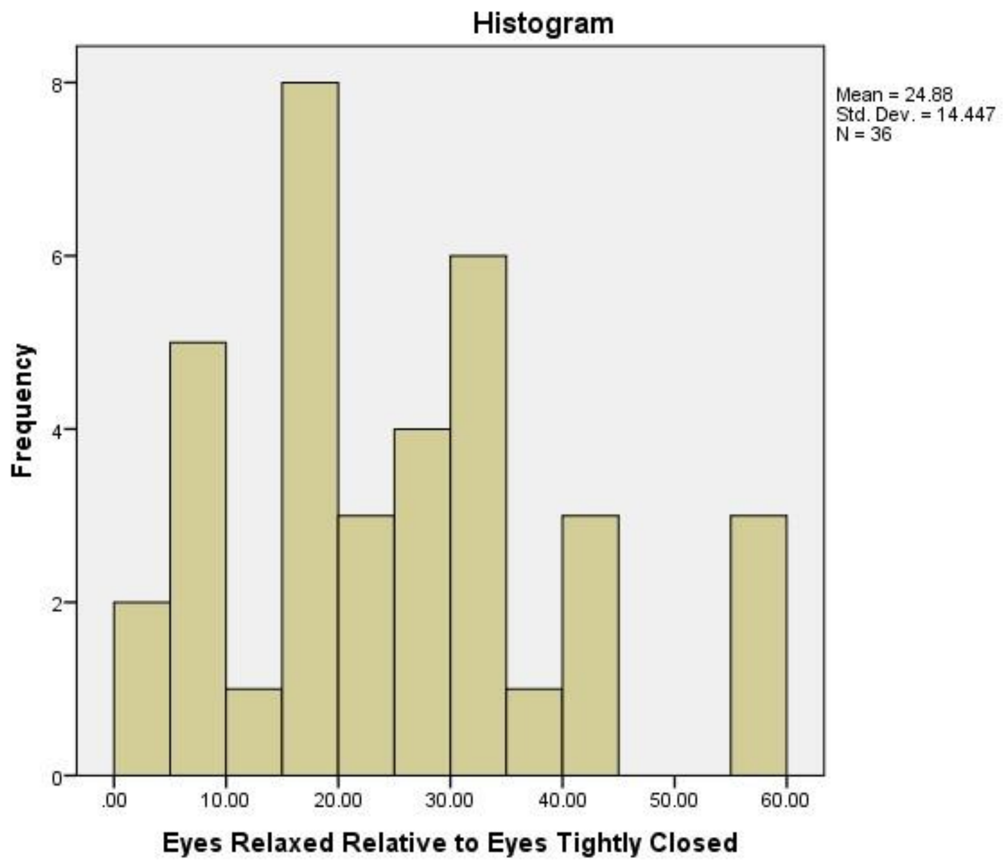

Figure S1. A histogram of the paired differences between the nasal valve cross-sectional area in the “relaxed” and “eyes tightly closed” positions.

Table S1: Results of Shapiro-Wilk normality test of the normalized data per paired differences.

Normalized Condition	Shapiro-Wilk		
	**Statistic**	**DF**	**P-value**
**Relaxed vs Closed**	0.951	36	0.116
**Open vs Closed**	0.782	36	<0.001
**Open vs Relaxed**	0.920	36	0.013

Table S2: Descriptive statistics of the data normalized with regard to "relaxed" position

Group	Case	Min	P25	Mean CI 95%	Std	Median	P75	Max
**All Sample N = 36**	**Closed**	43.35	66.37	75.12 (80.01, 70.23)	14.44	75.61	84.58	97.76
	**Open**	69.32	89.65	100.80 (106.75, 94.85)	17.59	98.04	111.42	164.62
**Females N = 27**	**Closed**	43.35	65.59	73.48 (79.35, 67.62)	14.82	75.38	84.11	95.64
	**Open**	69.32	89.65	101.24 (108.73, 93.76)	18.92	98.65	112.97	164.62
**Males N = 9**	**Closed**	59.55	69.67	80.02 (89.82, 70.22)	12.74	82.12	92.13	97.76
	**Open**	80.11	89.13	99.47 (110.02, 88.92)	13.72	95.50	110	121.75

Table S3: Mann-Whitney U Test results

Condition	Median Difference (Normalized Units)	Statistic	*p*-value
Eyes Tightly Closed	6.746	Z=0.932	0.352
Eyes Wide Open	3.156	Z=0.311	0.756
